# Postnatal maturation of the spinal-bulbo-spinal loop: brainstem control of spinal nociception is independent of sensory input in neonatal rats

**DOI:** 10.1097/j.pain.0000000000000420

**Published:** 2015-11-13

**Authors:** Fred Schwaller, Charlie Kwok, Maria Fitzgerald

**Affiliations:** Department of Neuroscience, Physiology & Pharmacology, University College, London, United Kingdom

**Keywords:** Neonatal pain, Descending control, Dorsal horn, Ascending pathways, Parabrachial nucleus, Periaqueductal gray, Rostroventral medulla, Receptive field

## Abstract

The rostroventral medial medulla (RVM) is part of a rapidly acting spino-bulbo-spinal loop that is activated by ascending nociceptive inputs and drives descending feedback modulation of spinal nociception. In the adult rat, the RVM can facilitate or inhibit dorsal horn neuron inputs but in young animals descending facilitation dominates. It is not known whether this early life facilitation is part of a feedback loop. We hypothesized that the newborn RVM functions independently of sensory input, before the maturation of feedback control. We show here that noxious hind paw pinch evokes no fos activation in the RVM or the periaqueductal gray at postnatal day (P) 4 or P8, indicating a lack of nociceptive input at these ages. Significant fos activation was evident at P12, P21, and in adults. Furthermore, direct excitation of RVM neurons with microinjection of DL-homocysteic acid did not alter the net activity of dorsal horn neurons at P10, suggesting an absence of glutamatergic drive, whereas the same injections caused significant facilitation at P21. In contrast, silencing RVM neurons at P8 with microinjection of lidocaine inhibited dorsal horn neuron activity, indicating a tonic descending spinal facilitation from the RVM at this age. The results support the hypothesis that early life descending facilitation of spinal nociception is independent of sensory input. Since it is not altered by RVM glutamatergic receptor activation, it is likely generated by spontaneous brainstem activity. Only later in postnatal life can this descending activity be modulated by ascending nociceptive inputs in a functional spinal-bulbo-spinal loop.

## Abstract 2

In young rats, below 12 days old, the rostroventral medulla exerts a tonic descending facilitation of spinal nociception, independent of ascending sensory input.

## 1. Introduction

Spinal dorsal horn sensory inputs and nociceptive reflexes are modulated by descending brainstem controls^45^ that underlie the control of pain by stress, fear, reward, and expectation.^6,13,25,44^ The rostroventral medulla (RVM) plays a key role in this process as it contains neurons that project down to the spinal dorsal horn^27,30^ and receive inputs from higher centers such as the insular cortex,^47^ the periaqueductal gray (PAG), and parabrachial (PB) nucleus.^8,39,55^ Nociceptive dorsal horn pathways terminate in the PB nucleus and PAG (the afferent limb), which in turn activate RVM neurons projecting back to the dorsal horn (the efferent limb) forming a spinal-bulbo-spinal loop. Descending RVM modulation in adult animals is biphasic and can facilitate or inhibit acute spinal nociception^16,17,60^ in an injury and context-specific manner.^10,38,51,60^

In young animals, dorsal horn tactile and nociceptive circuits are shaped by activity-dependent mechanisms.^18,20,32^ Periaqueductal gray–RVM modulation of spinal nociceptive circuits is slow to mature and is predominantly facilitatory until 4 weeks after birth,^21,22,33,34^ rendering the system vulnerable to changes in noxious sensory inputs.^9,52^ However, little is known about the postnatal development of a functional spinal-bulbo-spinal loop. We hypothesize that, in the early postnatal period, descending RVM control of spinal nociception occurs in the absence of ascending sensory inputs. Only later in life does the formation of a spinal-bulbo-spinal loop allow sensory modulation of descending controls. We tested this using in vivo functional mapping and dorsal horn electrophysiology together with direct excitation and silencing of the RVM at different postnatal ages.

## 2. Methods

### 2.1. Animals

All experiments were performed in accordance with the United Kingdom Animal (Scientific Procedures) Act 1986. Reporting is based on the Animal Research: Reporting of In Vivo Experiments guidelines developed by the National Centre for Replacement, Refinement, and Reduction of Animals in Research, London, United Kingdom.^29^ Male and female Sprague-Dawley rats at various ages from postnatal day (P) 4 to P45 were obtained from the Biological Services Unit, University College London.

Rats were bred and maintained in house and exposed to the same caging, diet, and handling throughout development. Litters were weaned at P21 into same sex cages of 4 littermates and were housed in 12 hours light/dark cycles at constant ambient temperature and humidity with free access to water and food.

### 2.2. Electrophysiology

Rats were anesthetised with isoflurane (induction 4% in medical O_2_), tracheotomized, and artificially ventilated under constant isoflurane anesthesia (maintenance of 1.8% in medical O_2_, Univentor Anaesthesia Unit 400; Royem Scientific, Luton, United Kingdom). The air flow and breathing rate were adjusted to the animal's sizes using a small animal ventilator (model 687; Harvard Apparatus, MA). Heart rate was constantly monitored by electrocardiogram. A homeothermic blanket with feedback control (model 507220F; Harvard Apparatus) and heating lamp were used to maintain body temperature at physiological levels. The rat was mounted onto a stereotaxic frame (Kopf Instruments, Tujunga, CA). A laminectomy was performed to expose the lumbar spinal cord, the vertebral column was secured with a clamp to the thoracic site and the dura and pia mater were removed. A film of mineral oil was used to cover the exposed spinal cord to prevent heat loss. The skull was exposed and bregma located to perform a small craniotomy for RVM microinjection.

### 2.3. Rostroventral medulla stimulation and silencing

Stereotaxic coordinates for the RVM were calculated as outlined previously.^21^ A 26-gauge 10-μL syringe (Hamilton, Reno, NV) was lowered into the RVM and drug or saline was injected over a 5-minute period. The experimenter was blinded to the drug administered. At the end of experiments, animals were terminally anesthetized with an intraperitoneal injection of an overdose of pentobarbitone (Euthatal; United Kingdom). The brain was dissected out to allow visual inspection of the injection site. Data from animals with injection sites which lay outside the RVM were rejected.

In RVM excitation experiments, the excitatory glutamatergic analog DL-homocysteic acid (DLH; nonselective N-methyl-D-aspartate receptor agonist; Sigma-Aldrich, United Kingdom) was dissolved in Ringer solution and saline to a concentration of 10 mg/mL (1%, pH 6.9). Of note, 0.7 to 1 μL of injectate containing 7 to 10 μg (depending on age) of DLH was microinjected into the RVM. In RVM silencing experiments, lidocaine hydrochloride monohydrate (Sigma-Aldrich, United Kingdom) was dissolved in saline to a concentration of 20 mg/mL (2%, pH 5.5). Of note, 0.7 to 1 μL of injectate containing 14 to 20 μg of lidocaine was microinjected into the RVM. Control animals received equivalent volumes of saline.

### 2.4. In vivo extracellular recordings in the dorsal horn

To isolate individual neurones in the dorsal horn, a 6-μm tipped glass-coated carbon fiber microelectrode (Kation Scientific, Minneapolis, MN) was lowered through the cord in 2 to 10 μm steps with a microdrive (Digitimer SCAT-01 microelectrode stepper system; Digitimer, United Kingdom) while stroking the plantar surface of the hind paw as a search stimulus for dorsal horn wide dynamic range (WDR) cells in lamina IV-VI. Cutaneous receptive fields to brush and pinch stimulation were mapped and the number of spikes per stimulus to brush, pinch, and von Frey hair (vFh) stimulation of the receptive field were recorded. Brush and vFh stimuli were applied for 0.5 seconds and pinch stimulation for 2 seconds. To avoid sensitization of nociceptors in younger animals, the maximum vFh force applied to P8-10 rats was 6.7 g and to P21 and adult rats, it was 9.8 g. Stimulus-evoked potentials were digitalized using PowerLab 4/30 interface and isolated using the Chart 5 software spike histogram plug-in (AD Instruments Ltd, Oxford, United Kingdom).

In RVM activation experiments, one cell was recorded per animal (P10 n = 19; P21 n = 17; and adult n = 18). A WDR neuron was isolated and recorded from to establish baseline cell receptive fields and brush, pinch, and vFh-evoked firing properties. DL-homocysteic acid was then microinjected into the RVM and the same train of peripheral stimuli was applied to the hind paw receptive fields 20 minutes after injection to allow within-cell changes to be analyzed. A previous study has demonstrated that the maximum effect of glutamate analog injection is up to 30 minutes after RVM injection.^27^

In RVM silencing experiments, lidocaine or saline was microinjected into the RVM. Wide dynamic range neuron recordings were performed 10 minutes after RVM microinjection up to 90 minutes; a timeframe which is within the period of maximal effect of lidocaine.^3^ Cell properties were compared as populations from lidocaine-treated animals (P8 = 22 cells from 4 animals; P21 = 24 cells from 4 animals; and adult = 17 cells from 4 animals) and control animals (P8 = 21 cells from 6 animals; P21 = 28 cells from 4 animals; and adult = 23 cells from 7 animals). The control cell population is a pooled group of cells from animals receiving RVM saline (P8 = 7 cells from 2 animals; P21 = 15 cells from 2 animals; and adult = 13 cells from 2 animal) and naive animals which displayed the same cell properties.

### 2.5. Cutaneous pinch stimulation and fos immunohistochemistry

Animals at P4, P8, P12, P21, and P40 (n = 4 per age) were anaesthetized with isoflurane and maintained at a low level of anesthesia sufficient to cause areflexia (1.8%-2%). The pinch stimulus was applied with forceps for 5 seconds on 6 points on both the dorsal and ventral surface of the left hind paw over the course of 1 minute as previously described.^43^ Control animals (n = 4 per age) received the same length of anesthesia.

Two hours after pinch stimulation/anesthesia, rats were reanesthetized with pentobarbitone sodium (500 mg/kg) and perfused transcardially with heparinised saline (5000 IU/mL) followed by 4% paraformaldehyde in 0.1 M phosphate buffer. The brain was removed and postfixed overnight in 4% paraformaldehyde and transferred to a 30% sucrose solution in 0.1 PB containing 0.01% azide and stored at 4°C. Periaqueductal gray, PB, and RVM tissue were collected in 40 μm transverse sections.

Brainstem sections were double labeled for c-fos and NeuN. For c-fos staining, free-floating sections were blocked with 3% goat serum in 0.3% Triton X-100 in 0.1 M phosphate buffer solution for 1 hour at room temperature. Sections were then incubated overnight at room temperature with rabbit anti–c-fos antibody (1:20,000; Millipore, Darmstadt, Germany). The next day, sections were incubated in biotinylated antirabbit antibody (goat antirabbit; 1:400; Vector Stain) for 90 minutes. Sections were placed in ABC complex (1:125; Vector Stain, ABC elite kit; Vector Labs, Burlingame, CA) for 30 minutes, followed by biotinylated tyramide (1:75; TSA Stain Kit; Perkin Elmer, Waltham, MA) for 7 minutes. Sections were then incubated in fluorescence isothiocyanate (FITC; 1:600; Vector Stain) for 2 hours. For double labeling with NeuN, sections were incubated in mouse anti-NeuN (1:500; Chemicon, Temecula, CA) overnight before a final 2-hour incubation with goat antimouse Alexafluor 594 (1:250; Invitrogen, Carlsbad, CA).

### 2.6. Fos-immunoreactivity cell counting

In immunohistochemistry experiments, the presence of Fos-immunoreactive (Fos-ir) neurons was examined in the ventrolateral PAG (vlPAG), the PB nucleus, and the RVM. In the PB area, the spinocerebellar tract, the inferior colliculus, and the brachium conjunctivum were used as landmarks and rostrocaudal distribution of sections were determined with respect to the reference plane where the inferior colliculus merges with the pons. Fos-immunoreactive neurons were counted in the contralateral mesencephalic PBel and PBsl and the pontine PBel; areas which are known to receive ascending input from the superficial dorsal horn in adult studies.^11,14,48^ In the PAG, Fos-ir neurons were examined in the contralateral ventrolateral region which receives ascending input from the spinal dorsal horn^28^ and includes neurons that directly project to the RVM^55^ in adult animals. In the RVM, Fos-ir cells were counted in the nucleus raphe magnus, lateral paragigantocellular nucleus, and gigantocellular reticular nucleus alpha.

### 2.7. Microsphere injection and visualization

Newborn (P0, n = 4) and P6 (n = 4) rats were anaesthetized with isoflurane. A laminectomy at lumbar level 4 to 5 was performed and the dura was incised to expose the spinal dorsal horn. Microspheres (Retrobeads, Lumafluor, Durham, NC) were injected into the lumbar dorsal horn using a 2.5-μL Hamilton syringe fitted with a 28G micropipette. Of note, 0.2 μL of microsphere solution was injected into the left dorsal horn, after which the pipette tip was held in the injection site for 1 minute before withdrawal. The skin was then sutured with 5 to 0 suture (Ethicon, Somerville, NJ), and Eutectic Mixture of Local Anesthetics cream (AstraZeneca, London, United Kingdom) was applied to the wound before the pups were returned to their cage.

Animals were perfused as described above 4 days after intraspinal injection of microspheres (Retrobeads; Lumafluor). Spinal cord and RVM tissue was taken and collected in 40 μm transverse sections before blocking in 3% goat serum, incubation overnight with mouse NeuN (1:500), and fluorescent marking for 2 hours with goat antimouse Alexafluor 594 (1:250).

All sections were mounted on gelatinized slides and were then cover slipped with Fluoromount (Sigma-Aldrich). Negative control stains omitting primary antibodies resulted in no immunofluorescence, demonstrating no nonspecificity of any protocol. Sections were viewed using a Leica digital module R light microscope, photographed using a Hamamatsu C4742-95 digital camera and analyzed with Volocity software 6.3.

### 2.8. Statistical analyses

Statistical analyses and graphing were performed using GraphPad Prism 6 (GraphPad software, La Jolla, CA), and *P* < 0.05 was considered statistically significant. Sample sizes for testing were based on previously reported group differences between RVM silenced/stimulated animals^3,33,53^ and Fos immunohistochemistry experiments.^2,5^ All datasets were normally distributed, therefore parametric statistical tests were used. Data are represented as means ±SEM.

In immunohistochemistry experiments, Fos-ir neurons were counted in 4 to 5 sections per animal, which were averaged to create one value per animal. The mean pinch-induced Fos-ir and mean control Fos-ir in each brainstem region were compared at each age using 2-way analysis of variance (ANOVA) followed by Bonferroni post hoc multiple comparisons test.

In DLH electrophysiology experiments, single neurons were followed before and after RVM injection. Neurons were pooled from each age and group differences at baseline and 20 minutes after DLH injection tested using paired Student *t* tests. Individual cells are shown as facilitated, inhibited, or not changed after injection, defined as a >10% increase or decrease in firing rate or receptive field size over baseline. For vFh responses, a 2-way repeated measured ANOVA were used within the age groups followed by Bonferroni post hoc multiple comparison, where vFh force and time points (baseline and 20 minutes after intra-RVM DLH microinjection) were variables.

In RVM lidocaine electrophysiology experiments, WDR neurone recordings from lidocaine-treated animals were pooled and treated as one population of neurones for each age. Data from naive animals were combined with data from RVM saline–treated animals as a control group, as there were no statistical differences between them at any age (data not shown). Group differences between control and lidocaine-treated animals within age groups were tested with unpaired Student *t* tests and one-way ANOVA followed by Bonferroni post hoc multiple comparison tests. In vFh experiments, 2-way ANOVA was used within the age groups followed by Bonferroni post hoc multiple comparison test to compare differences in responses to increasing vFh force in control animals and lidocaine-treated animals.

Dorsal horn neuron receptive fields were drawn on a template during recording and then imported and expressed as a percentage of the total area of the hind paw plantar surface using Inkscape (version 0.48; www.inkscape.org).

## 3. Results

### 3.1. Functional nociceptive inputs to the periaqueductal gray and rostroventral medulla form at P12

**Figure 1:**
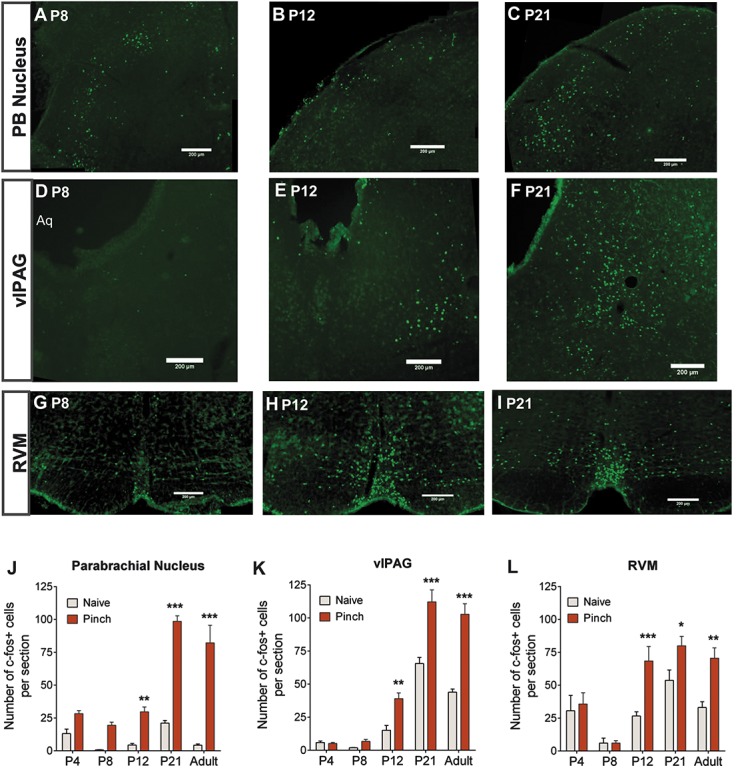
Noxious sensory input into the parabrachial (PB) nucleus, the ventrolateral periaqueductal gray (vlPAG), and the rostroventral medulla (RVM) is not evident before postnatal day (P) 12. Hind paw pinch stimulation causes substantial fos expression in the PB at P8 (A), P12 (B), and P21 (C). In comparison, pinch-induced fos expression was low in the vlPAG at P8 (D) but was significant at P12 (E) and even more so at P21 (F). Similarly, pinch-induced fos expression was sparse in the RVM at P8 but was significantly increased at P12 (H) and P21 (I). Quantification of naive and pinch-induced fos expression in the PB nucleus demonstrated significantly higher fos counts in pinch animals compared with naive animals at P12, P21, and in adults (J). Quantification in the vlPAG (K) and RVM (L) also demonstrated significantly higher fos counts in pinch animals compared with naive animals at P12, P21, and in adults. Two-way analysis of variance with Bonferroni post hoc analysis *, **, *** *P* , 0.05, 0.01, and 0.001 pinch compared with naive within each age. Note the high fos expression in the RVM in naive and pinch animals alike at P4 (L). Scale bars = 200 μm.

To test the age at which ascending functional nociceptive connections are formed in the brainstem, and the afferent limb of the spinal-bulbo-spinal loop is established, noxious-evoked fos activity was mapped in 3 brainstem regions: the PB nucleus, vlPAG, and the RVM (Figs. 1A–I). Noxious pinch stimulation of the hind paw under light anesthesia was applied at different ages from P4 to P40 and the number of neurons expressing fos in the 3 sites was counted. Figure 1J shows that in the PB nucleus, the number of Fos-ir cells significantly increased after noxious hind paw pinch stimulation compared with control at P12, P21, and in adults (2-way ANOVA with Bonferroni post hoc analysis, control vs pinch, *P* < 0.05-0.001 at different ages). Although a 2-way ANOVA with Bonferroni post hoc comparison did not reveal significance at P4 and P8, unpaired Student *t* tests did demonstrate significantly increased Fos-ir after pinch compared with control (Fig. 1J). There was no fos increase in the vlPAG and the RVM after hind paw, noxious pinch stimulation at P4 or P8 but a significant increase in the number of Fos-ir cells compared with control at P12, P21, and in adults (2-way ANOVA with Bonferroni post hoc analysis, control vs pinch, *P* < 0.05-0.001 at different ages, Figs. 1K and L). These findings suggest that the PAG and the RVM system do not receive noxious ascending input until P12, however the PB nucleus receives this input earlier within the first postnatal week.

### 3.2. Direct excitation of rostroventral medulla neurons does not alter dorsal horn cell activity at P10

**Figure 2:**
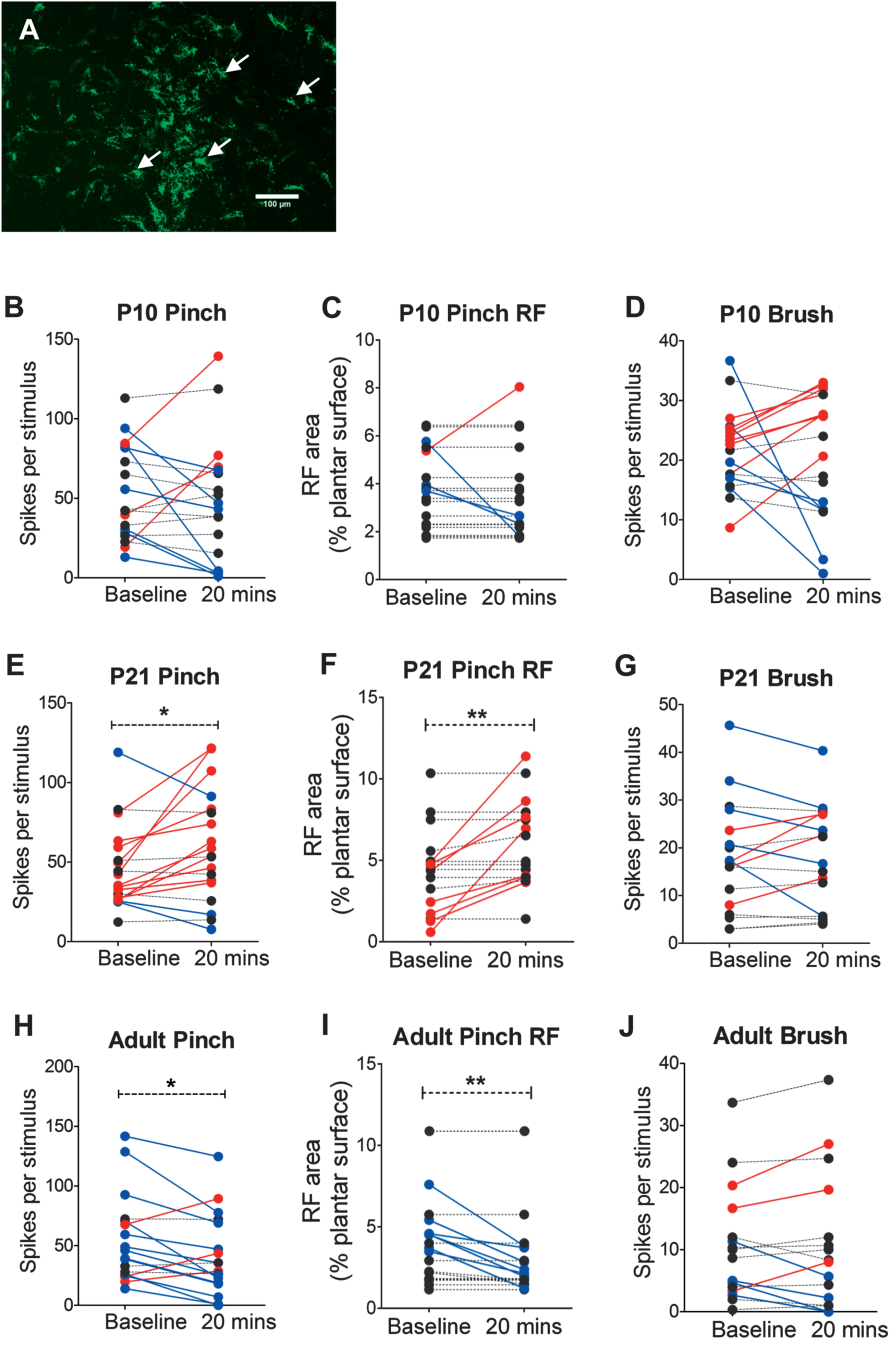
Direct excitation of rostroventral medulla (RVM) neurons by microinjection of DL-homocysteic acid (DLH) has alters dorsal horn neuron activity at postnatal day (P) 21 and adulthood but not at P10. (A) Intraspinal injections of microspheres at P0 retrogradely labels neurons in the RVM 4 days after injection, demonstrating that RVM neurons sent axons to the cord from an early age. Scale bars = 200 μm. (B–J) The effect of microinjection of DLH into the RVM on the activity and receptive field properties of wide dynamic range (WDR) dorsal horn neurons in L4/5. For illustrative purposes, cells in which RVM DLH injection caused an increase in firing rate or receptive field (RF) size compared with baseline (>10%) are shown in red, cells in which there was a decrease in firing rate or RF size are shown in blue, cells with no change are in black. At P10, RVM DLH injection did not change WDR neuron pinch-evoked firing rate (B) and pinch RF size (C) or brush-evoked firing rate (D) compared with baseline. At P21, mean WDR neuron pinch-evoked firing rate was higher (E) and RF size was larger (F) 20 minutes after RVM DLH injection compared with baseline. Brush-evoked firing rate was not changed after RVM DLH injection (G). In adult animals, RVM DLH injection decreased pinch-evoked firing rate (H) and pinch RF size (I) compared with baseline. Brush-evoked firing was not changed after RVM DLH injection (J). Paired Student *t* test *, **P < 0.05 and 0.01 RVM DLH microinjection +20 minutes compared with predrug baseline WDR neuron responses.

We next turned to the descending limb of the spino-bulbar-spinal loop by testing whether direct excitation of RVM neurons was able to alter dorsal horn neuron firing properties at young rat pups. First, we confirmed that RVM neurons project to the lumbar spinal dorsal horn in newborn rats with retrograde labeling with intraspinal microinjection of microspheres. Numerous neuronal cell bodies were retrogradely labeled in the RVM at P4 and P10 after intraspinal microinjection of microspheres into the L4/5 dorsal horn at P0 and P6, respectively (Fig. 2A).

Next, we tested the age at which direct glutamatergic excitation of RVM neurons can alter dorsal horn cell activity by microinjecting the glutamate analog DLH into the RVM and measuring the effect on the properties of spinal WDR neurons at different ages. Receptive field sizes and stimulus-evoked spike activity were recorded before and 20 minutes after DLH microinjection into the RVM in P10 (n = 19), P21 (n = 17), and adult (n = 18) rats.

In P10 rats, the mean pinch-evoked spike activity of WDR neurons was not significantly different 20 minutes after DLH compared with baseline (paired *t* test, baseline vs 20 minutes, *P* = 0.44; Fig. 2B). Similarly, pinch receptive field size was not significantly different 20 minutes after DLH compared with baseline (paired *t* test, baseline vs 20 minutes, *P* = 0.46; Fig. 2C). In P21 rats, RVM DLH microinjection increased the mean pinch-evoked spike activity (paired *t* test, baseline vs 20 minutes, *P* < 0.05; Fig. 2E) and pinch receptive field size (paired *t* test, baseline vs 20 minutes, *P* < 0.01; Fig. 2F). In contrast, RVM DLH microinjection in adults decreased the mean pinch-evoked spike activity (paired *t* test, baseline vs 20 minutes, *P* < 0.05; Fig. 2H) and pinch receptive field size (paired *t* test, baseline vs 20 minutes, *P* < 0.01; Fig. 2I). It should be noted that these mean values reflect “net” changes in the dorsal horn but that the effects on individual dorsal horn neurons varied (Fig. 2).

Rostroventral medulla DLH microinjection did not change brush-evoked firing activity (paired *t* test, baseline vs 20 minutes, *P* > 0.05; Figs. 2D, G and J) or brush receptive field sizes (data not shown) at any age tested. Similarly, DLH microinjection did not change spontaneous firing activity at any age tested (paired *t* test, baseline vs 20 minutes, *P* > 0.05; data not shown). These data demonstrate that direct exogenous glutamatergic activation of RVM neurons does not modulate spinal WDR neuron nociception at P10 but does do so at P21 and in adult rats. At P21, RVM neurons facilitate spinal nociception, whereas in adults, they inhibit nociception.

### 3.3. The rostroventral medulla exerts a tonic facilitation on dorsal horn neurons from at least P8

The above experiments suggest that before P12, there is no functional nociceptive input to RVM neurons nor is it possible to modulate dorsal horn activity on exogenous excitation of RVM neurons. This suggests that the RVM is not part of a spino-bulbo-spinal loop at this age. However, it is still possible that immature RVM neurons provide tonic descending input to the dorsal horn in the absence of active afferent inputs. We tested this by silencing RVM neuron activity with microinjection of lidocaine into the RVM of P8, P21, and adult rats and measuring the effect on spinal dorsal horn WDR neuron activity. Cutaneous receptive fields and evoked spike activity of WDR neurons were recorded in 2 groups of animals, RVM lidocaine animals (P8, n = 22 from 4 animals; P21, n = 24 from 4 animals; and adult, n = 17 from 4 animals) and control animals (P8, n = 21 from 6 animals; P21, n = 28 from 4 animals; and adults, n = 23 from 7 animals) in response to brush, pinch, and vFh stimulation.

**Figure 3:**
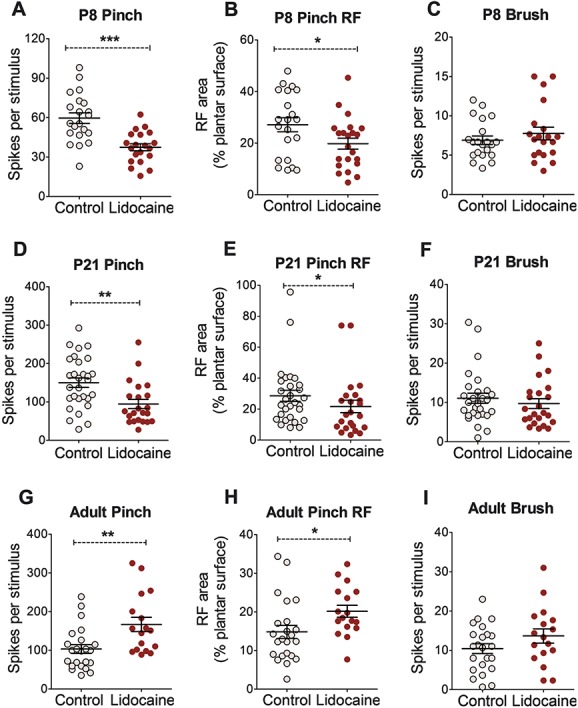
Focal microinjection of lidocaine in the rostroventral medulla (RVM) alters pinch-evoked dorsal horn cell activity at all ages. At postnatal day (P) 8, RVM lidocaine injection reduced the mean wide dynamic range (WDR) neuron pinch-evoked firing rate (A) and pinch receptive field (RF) size (B) but not brush-evoked firing rate (C) compared with age-matched control. Much the same occurred at P21 (D–F). In adult animals, RVM lidocaine injection increased pinch-evoked firing rate (G) and pinch receptive field size (H) but did not change brush-evoked firing rate (I) compared with control. Unpaired Student *t* test *, **, ***P < 0.05, 0.01, and 0.001 compared with age-matched control WDR neuron population responses.

In P8 animals, the mean number of spikes per pinch stimulus in RVM lidocaine animals was significantly lower than control animals (unpaired Student *t* test, *P* < 0.001; Fig. 3A). The mean pinch receptive field size was also smaller in RVM lidocaine animals compared with control animals at P8 (unpaired Student *t* test, *P* < 0.05; Fig. 3B). A similar effect of silencing the RVM was observed at P21. Mean pinch-evoked firing activity was significantly lower and mean pinch receptive field size was significantly smaller in RVM lidocaine animals compared with control animals (unpaired Student *t* test, *P* < 0.01 and *P* < 0.05, respectively; Figs. 3D and E). In adults, the reverse pattern was observed: the mean pinch-evoked firing activity was significantly higher and the mean pinch receptive field size was significantly larger in RVM lidocaine animals compared with control animals (unpaired Student *t* test, *P* < 0.01 and *P* < 0.05, respectively; Figs. 3G and H). Rostroventral medulla lidocaine microinjection did not significantly affect brush-evoked WDR spike activity (Figs. 3C, F and I) or brush receptive field sizes (data not shown) compared with control at any age tested. Similarly, RVM lidocaine microinjection did not significantly affect spontaneous firing activity when compared with control at any age (unpaired Student *t* test, *P* > 0.05; data not shown).

**Figure 4:**
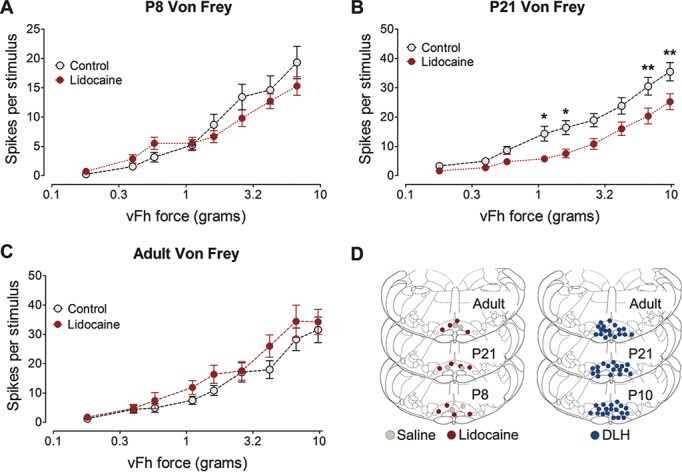
Focal microinjection of lidocaine into the rostroventral medulla (RVM) has age-dependent effects on von Frey hair (vFh) stimulus response relationships in dorsal horn neurons. (A) At postnatal day (P) 8, RVM lidocaine injection did not change vFh-evoked firing rates at any force applied. (B) At P21, RVM lidocaine injection reduced wide-dynamic-range (WDR) firing rates when 1.1, 1.6, 6.7, and 9.8 g vFhs were applied, when compared with control responses. (C) In adult animals, RVM lidocaine injection did not change vFh-evoked firing rates at any fore applied compared with control. Two-way analysis of variance with Bonferroni post hoc analysis *, **, ***P < 0.05, 0.01, and 0.001 compared with age-matched control WDR neuron population responses. Injection sites for each age for RVM lidocaine and RVM DL-homocysteic acid experiments are shown in (D).

Next, calibrated vFhs were applied to hind paw receptive fields of WDR neurons in RVM lidocaine animals and control animals. Comparison of stimulus response curves between RVM lidocaine and control animals at P8 revealed no significant effect of RVM lidocaine microinjection on vFh-evoked firing activity (2-way ANOVA with Bonferroni post hoc analysis, control vs lidocaine, F_1,29_ = 0.378, *P* = 0.543; Fig. 4A). At P21, vFh-evoked firing activity was significantly lower in RVM lidocaine animals than control animals (2-way ANOVA, control vs lidocaine, F_1,47_ = 8.488, *P* < 0.01; Fig. 4B). Bonferroni post hoc analysis revealed significant differences between RVM lidocaine and control cells at 1.1, 1.6, 6.7, and 9.8 g vFhs (2-way ANOVA with Bonferroni post hoc analysis, control vs lidocaine, *P* < 0.01-0.05 at different vFh forces; Fig. 4B). In adult animals, RVM silencing did not alter vFh-evoked firing activity compared with control (2-way ANOVA with Bonferroni post hoc analysis, control vs lidocaine, F_1,35_ = 1.361, *P* = 0.251; Fig. 4C).

These data demonstrate that at P8, there is a background, tonic RVM facilitation of noxious pinch responses of spinal WDR neurons. This facilitation is also present at P21 but is more extensive and has changed to inhibition in adult animals.

## 4. Discussion

The data presented here support the hypothesis that the efferent, descending limb of the spinal-bulbo-spinal loop arising from the RVM is functionally active at P8, before RVM neurons are influenced by ascending inputs. In this early postnatal period, RVM descending control on dorsal horn neurons is independent of noxious sensory input to the RVM. At P8, silencing RVM neurons by focal injection of lidocaine revealed a facilitatory role of the RVM on spinal dorsal horn neurons, but interestingly, this descending control did not require ascending or exogenous recruitment of the RVM, as hind paw pinch stimulation failed to increase c-fos expression in the vlPAG and RVM at P8, and glutamate stimulation of the RVM at P10 failed to modulate spinal dorsal horn neuron activity. In contrast, RVM descending control at P21 and in adult rats can be driven by RVM neuron activation; be it as part of a functional spino-bulbo-spinal loop by peripheral noxious stimulation or by focal application of a glutamate analog. We also show, as described previously, that net RVM descending control over dorsal horn neurons is facilitatory in young animals and inhibitory in adult animals but targets nociceptive input at all ages. These conclusions are limited to cutaneous mechanical sensory inputs, but these are especially relevant to normal postnatal life.

Endogenous descending control of nociceptive activity is an important aspect of central nervous system pain processing. In adults, a change in balance of excitatory and inhibitory drive from the RVM can strongly modulate spinal dorsal horn sensory inputs in acute and chronic pain states. A rapid feedback loop, driven by ascending pathways from the spinal dorsal horn and acting through the PAG-RVM, is vital in moment-to-moment modulation of nociceptive inputs.^49^ The apparent dichotomy of facilitation and inhibition is not a simple “on–off” mechanism, but rather a system where inhibition and facilitation can operate in parallel to produce a balanced net outcome which is appropriate to a particular situation.^10,50,51,56^ In young animals, this balance is not yet achieved and descending output is predominantly facilitatory.

Here, we silenced neural activity in the RVM (neurons and fibers of passage) by focally injecting lidocaine into the RVM at different ages to investigate the endogenous and ongoing role of the RVM in modulating dorsal horn sensory inputs during postnatal development. At P8 and P21, silencing the RVM-reduced pinch-evoked dorsal horn neuron excitability, demonstrating that in young animals the RVM exerts a net facilitation of nociceptive inputs onto dorsal horn WDR neurons. This is consistent with lesions to the RVM which unmask descending facilitation of behavioral nociception as young as P3.^21^ Silencing of RVM neurons by focal injection of lidocaine has previously been shown to inhibit activity in some and facilitates activity in most individual neurons in adult rats,^3^ however we found that silencing the RVM in uninjured adult rats increased net dorsal horn neuron excitability, unmasking a net inhibition of spinal nociception. Others have used this type of overall output analysis to show reduced injury-induced behavioral hypersensitivity after PAG-RVM silencing with lidocaine, demonstrating a net descending facilitation during pain states.^10,31,40^

Electrical stimulation of descending brainstem nuclei has been extensively used to investigate functional control over dorsal horn sensory inputs^23,45^; however, nonselective activation of neighboring neurons and fibers of passage can confound results. In this study, we selectively activated RVM neurons by injecting the glutamate analog DLH into the RVM and compared individual dorsal horn neuronal changes before and after injection. DL-homocysteic acid stimulation of the RVM at P21 strongly increased pinch-induced excitability of most dorsal horn neurons and DLH stimulation of the RVM in adult animals decreased pinch-induced excitability of most dorsal horn neurons. In accordance with previous RVM electrical stimulation experiments,^15,21,33^ descending facilitation dominates at P21 and inhibition dominates in adults, however smaller population of neurons were also inhibited and facilitated at P21 and in adults, respectively. However, DLH stimulation of the RVM at P10 did not change dorsal horn neuron activity; although small subpopulations of dorsal horn neurons were inhibited or excited after peripheral stimulation; the excitability of most neurons was not changed by RVM stimulation, suggesting that the RVM neurons are not easily excited by exogenous stimulation at P10.

Peripheral noxious stimulation increases c-fos expression in the spinal cord and in nuclei which receive ascending nociceptive input such as the PB nucleus and the PAG.^4,26,28^ Neuronal activation, as measured by increased c-fos or phosphorylated extracellular signal-related kinase expression, has also been observed in the adult RVM after peripheral noxious stimulation or injury.^19,41,42^ Pinch and touch-induced c-fos expression has been reported in the dorsal horn at P3 and P10, and pinch-selective c-fos expression is apparent by P21.^26^ In contrast, formalin injection only increases c-fos expression in the PAG and thalamus at P14 and not at P3,^2^ suggesting later development of functional connections between the spinal dorsal horn and midbrain projection targets. Here, we show that hind paw pinch stimulation increases c-fos expression in the PB nucleus from P8. In contrast, pinch-induced c-fos expression only increased in the vlPAG and RVM after P12, suggesting that functional development of the ascending, afferent component of the spino-bulbo-spinal loop is later to mature than the descending component. Indeed, distal pinch stimulation can reduce spinal c-fos expression induced by heterotopic formalin injection at P21 but not at P12, suggesting functional diffuse noxious inhibitory control from P21 but not at P12.^7^ Moreover, injection of the μ-opioid receptor agonist [D-Ala2, N-MePhe4, Gly-ol]-enkephalin into the PAG fails to change spinal reflex excitability in P10 rats.^34^ These findings parallel the lack of overall modulation of dorsal horn neuron excitability after glutamatergic RVM neuron activation in P10 rats. Hence, adult-like recruitment of the spino-bulbo-spinal loop by peripheral noxious stimulation is not observed until the third postnatal week.

Despite this, our RVM silencing experiments at P8 demonstrated that there is an ongoing overall descending facilitation that does not require bottom-up or top-down recruitment, is seemingly selective to noxious inputs, and does not modulate the spontaneous activity of dorsal horn neurons. Without the capacity to be recruited by feedforward activation, the immature RVM would presumably be intrinsically active. Indeed, we observed fos activation in the RVM in the absence of noxious stimulation at P4 which may suggest the presence of spontaneously active neurons in the RVM at this age.^54^ Spontaneously firing “pacemaker” neurons have been well described in the neonatal rat spino-bulbo-spinal loop in the superficial spinal dorsal horn.^1,35,36^ These intrinsically bursting neurons may function to provide endogenous excitation of developing nociceptive circuits early in development. Although the presence of age-specific pacemaker neurons has not been identified in the neonatal RVM, patch-clamp experiments have demonstrated that most RVM neurons in adult brainstem slices do not exhibit spontaneous firing activity as frequently observed in RVM neurons in P10 to 21 brainstem slices.^37^ Several other studies have also demonstrated multiple subpopulations of locally and spinally projecting RVM neurons in brainstem slices of P21 or younger rats that readily display spontaneous firing activity,^57–59^ however few studies have directly compared RVM neuronal properties in young and adult animals. Spontaneous firing activity is not a property exclusive to neonatal RVM neurons, as baseline firing activity is a hallmark property of RVM “on” and “off” cells recorded from anesthetized and awake adult rats.^12,16,17,24^

In both the RVM glutamatergic activation and lidocaine silencing experiments, we demonstrate that the net function of the RVM is inhibitory in uninjured adult rats. These findings are in accordance with previous studies that have demonstrated a switch from net facilitation to inhibition between P21 and P40.^21,22,33,34^ This switch from facilitation from inhibition is dependent on sufficient levels of endogenous opioids in the RVM, as blocking opioidergic activity between P21 and P28 prevents the normal maturation of descending inhibition.^22^ Indeed, substantial changes in endogenous opioid signaling take place in the PAG and dorsal horn throughout development which may underlie the opioid-mediated maturation.^34^ Ingram and colleagues have identified late maturation of endocannabinoid control of RVM GABAergic neurotransmission,^37^ which could also contribute to maturation of descending inhibition. Additionally, brainstem dorsal raphe serotonergic neuron lack 5-HT_1A_ autoreceptor and gamma-aminobutyric acid-mediated inhibition until >P21, thus driving increased neuronal excitability in young rats.^46^ The data suggest that in the first 2 weeks of postnatal life, an intrinsic and ongoing descending drive from the RVM facilitates spinal dorsal horn sensory circuitry. This is likely to be of key importance in amplifying sensory inputs required for activity-dependent maturation of spinal circuitry. Moreover, amplified ascending connections from the dorsal horn may provide the excitatory drive required for maturation of the spino-bulbo-spinal loop and pain circuits in the brain.

**Figure 5:**
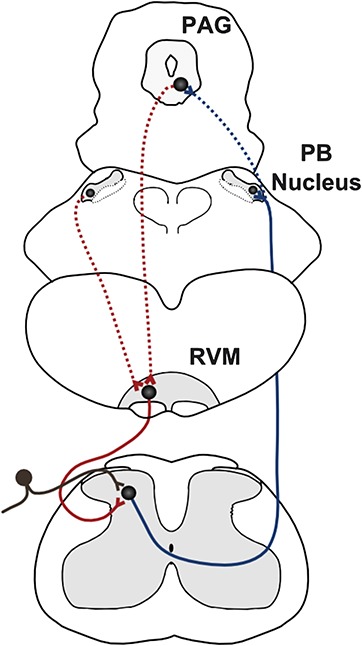
Proposed model of the delayed maturation of the spinal-bulbo-spinal loop. At postnatal day (P) 8-10, ascending nociceptive inputs from the dorsal horn (black) are beginning to activate neurons in the parabrachial (PB) nucleus (solid blue line). However, nociceptive connections to the periaqueductal gray (PAG) are not functional at this age (dotted blue line). The rostroventral medulla (RVM) projects to the spinal dorsal horn and modulates dorsal horn neuron activity (solid red line) but does not receive excitatory glutamatergic inputs from the PAG or elsewhere to complete the loop (dotted red lines). Therefore, at P8-10, descending RVM modulation of spinal dorsal horn activity acts independently of ascending sensory inputs.

Figure 5 illustrates our proposal that descending facilitatory control of spinal noxious activity in the early postnatal period arises from spontaneous activity within the RVM and only later in development can this descending activity be modulated by ascending sensory inputs in a spinal-bulbo-spinal loop. The delayed development of sensory feedback control is likely to render the immature nervous system more vulnerable to excessive sensory inputs or peripheral injury.

## Conflict of interest statement

The authors have no conflicts of interest to declare.

This work was funded by the Medical Research Council G0901269 (MF). F. Schwaller was supported by a UCL impact studentship.

F. Schwaller and C. Kwok contributed equally to this work.

## Supplemental media

Video content associated with this article can be found online as Supplemental Digital Content at http://links.lww.com/PAIN/A234.
